# Experimental Study of Bentonite-Free Water Based Mud Reinforced with Carboxymethylated Tapioca Starch: Rheological Modeling and Optimization Using Response Surface Methodology (RSM)

**DOI:** 10.3390/polym13193320

**Published:** 2021-09-28

**Authors:** Imtiaz Ali, Maqsood Ahmad, Tarek Ganat

**Affiliations:** 1 Department of Petroleum Engineering, Universiti Teknologi PETRONAS, Seri Iskandar 32610, Perak, Malaysia; 2 Department of Petroleum and Gas Engineering, BUITEMS, Quetta 87300, Balochistan, Pakistan; 3 Department of Petroleum and Chemical Engineering, Sultan Qaboos University, Muscat 123, Oman; t.ganat@squ.edu.om

**Keywords:** tapioca starch carboxymethylation, nondamaging water-based muds, rheological modeling, response surface methodology

## Abstract

Drilling mud’s rheological characteristics, such as plastic viscosity and yield point, are adversely affected with an inappropriate mud formulation. Native starch is one of the most important components in water-based mud because it improves the rheological and filtration characteristics of the mud. The native starch stability under various temperature and exposure time regimes is an important concern for utilizing starch in oil and gas drilling operations. In this work, tapioca starch was modified using carboxymethylation for the first time in order to improve its performance in non-damaging water-based muds. The modified starch was characterized by Fourier-transform infrared spectroscopy and X-ray diffraction. The thermal stability was tested using thermal gravimetric analysis. Various mud blends were formulated based on the experimental design using response surface methodology (RSM) to investigate their performance at various temperature conditions. Thirty experimental runs were carried out based on the selected factors and responses considering the optimal (custom) design, and the results were analyzed through ANOVA. The Fourier-transform infrared spectroscopy and X-ray diffraction results confirmed the carboxymethylation of starch. The TGA analysis revealed strong thermal stability after modification. Additionally, the Power law model (PLM) described the obtained rheological data for the selected formulations, resulting in determination coefficients of more than 0.95. Furthermore, the examined samples showed a reduction in the flow behavior index from 0.30 to 0.21 and an increase in the consistency index from 5.6 to 15.1. Optimization and confirmation results revealed the adequacy of the generated empirical models for both plastic viscosity and yield point. The obtained consistency index values provided a direct relationship with the modified starch concentration, indicating an improvement in the cutting carrying capacity of mud. Based on the current literature survey, the studied formulation has not been reported in the literature.

## 1. Introduction

Water based muds have been used in the oil and gas drilling industry for decades. A multi-functional drilling mud is required to perform numerous important functions including transporting cuttings, suspending cuttings, controlling formation pressure, cooling, and lubricating the bit, minimizing corrosion, providing necessary hydraulic power to down-hole equipment, and reducing formation damage. These functions can be achieved with mud exhibiting significant rheological and filtration properties. Rheological properties are influenced by various factors including solid content, pH, and additives such as dispersants, surfactants, and polymers [1,2,3,4].

The selection of proper mud additives is very important for a successful drilling operation. Numerous mud additives including viscosifiers, inhibitors, pH and fluid loss control agents are the primary additives used in water-based muds. For drilling the payzone section, a drilling fluid consists of polymers and other functionalized additives that are used to avoid the excessive fluid losses into the formation. Generally acid soluble materials including starches, polymers, and calcium carbonates are used to enhance the rheological and filtration properties of the mud [5,6]. Starch is an abundantly available semi-crystalline carbohydrate polysaccharide which has been used in oil and gas drilling for decades. Oil field applications of starch and its derivatives include fluid loss control, mud rheology improvement, shale stabilization, drag reduction, water shut-off, and oil recovery improvement [7].

The inadequate functional characteristics of native starches in water-based muds limit their applications owing to certain limitations such as their poor thermal stability, solubility, and bacterial resistance [8,9,10]. Thus, starches can be modified for various industrial applications due to the presence of abundant hydroxyl and hydrophilic groups [11]. The modification could affect the crystallinity, morphology, thermal stability, and hydrophobicity of the starch. Therefore, physical, chemical, enzymatic, or dual modifications are the promising approaches for developing new products, retaining petrochemical resources and enhancing specific starch properties [12]. Chemical modification is the most widely accepted approach and contributes to the emergence of new functionality to the starch. The added functionality could be any functional group that brings unique features to the starch.

In recent years, various starches have been modified chemically which substituted the imported drilling fluid additives. The modified starches exhibited better rheological and filtration properties in bentonite suspensions [10,13,14,15,16]. The better performance was attributed to the additional functional properties due to the existence of hydroxyl groups in the starch which is the primary reason that it provides more reactive sites for the chemical modification. Additionally, the structural differences impact the characteristics of modified starch, as alkalization will destroy the intermolecular bonds in the amorphous region of amylose, which tends to improve swelling properties. Acid-modified, pre-gelatinized, carboxymethylated, and hydroxy-ethylated starches have been used in bentonite-based muds that exhibited better rheological performance. Similarly, native starches have also been tested in drilling muds which showed improvements at standard and modest temperature conditions, but they lost their functionality when exposed to high saline and temperature conditions.

Out of all the methods, carboxymethylation is one of the most-used starch modification techniques in which the starch molecule’s -OH groups are partially substituted by the ether group (–OCH_2_COOH). It is a process in which starch hydroxyl groups react with monochloroacetic acid (MCA) or Monochloroacetate in the presence of a strong alkali. Several commonly used starches have been modified through carboxymethylation for different food and industrial applications. Carboxymethyl potato and corn starches have been employed largely for rheology improvements, filtrate control in drilling muds, drill-in, completion, and workover fluids [17,18]. From the mentioned studies, it has been noticed that the rheological and filtration characteristics of bentonite-based muds have been significantly improved with the native and modified starches. The complex network structure created between clay particles and starches enhanced the mud rheology and reduced the fluid loss [16].

It is important to point out that from the available literature survey, no study has been conducted wherein tapioca starch has been modified and utilized as a substitute of bentonite in non-damaging WBMs. Therefore, further investigation on the influence of functionalized starch on drilling fluid rheological characteristics under different temperature conditions is required. The aim of this study is to improve tapioca starch thermal stability by carboxymethylation to be used as a potential drilling fluid additive. The mud performance at high temperature conditions was evaluated in terms of plastic viscosity and yield point using API recommended standards. In addition, an ANOVA analysis was carried out, and empirical models were generated for the rheological parameters. Finally, the models were validated and optimized through additional experiments.

## 2. Experimental

### 2.1. Chemicals and Reagents

For carboxymethylation, monochloroacetic acid (MCA: purity ≥99%, Sigma Aldrich, Saint Louis, MO, USA) and sodium hydroxide (NaOH: Pellet, R&M chemicals) were used. Methanol (99%), ethanol (HMBG: 95%), and acetone (Merck, Kenilworth, NJ, USA) were used as solvents for modification purposes. Other additives including calcium carbonate (CaCO_3_: R&M chemicals), polyanionic cellulose (PAC: Scomi Oiltools, Dubai, UAE), and xanthan gum (XG: Scomi Oiltools, Dubai, UAE) were used for drilling mud formulations. All the materials were used as provided without further treatment.

### 2.2. Tapioca Starch Modification

Prior to the carboxymethylation of starch, native tapioca starch was dried in sunlight for 10 days followed by artificial drying at 50 °C for 48 h to eliminate the moisture content (≤10%). For carboxymethylation, a previous method by Rachtanapun, et al. [19] was followed with some modifications. Briefly, 50 g native tapioca starch were combined with 100 mL of deionized water and continuously stirred using a magnetic stirrer. Then, sodium hydroxide (dissolved in 100 mL of water) was introduced, preceded by more stirring for 1 h at 50 °C. The reaction is typically carried out in the presence of strong bases to enhance the nucleophilicity of the hydroxyl group and assist the swelling of the starch particles. Monochloroacetic acid (MCA) was mixed at varying ratios and stirred while it was heated for the mentioned time. The mixture was then allowed to cool until room temperature. Finally, the carboxymethylated tapioca starch (CMTS) was rinsed in 100 mL acetone, filtered, and dried in the oven at 50 °C for 24 h and stored.

### 2.3. Determination of Degree of Substitution (DS)

The number of functional groups introduced into the anhydro glucose unit (AGU) is defined by the degree of substitution. Since every AGU has four hydroxyl groups and the value of DS lies in the 0–3 range. Here, the degree of substitution was measured using the direct titration method, which is the most popular chemical assessment technique that can give an accurate assessment of the DS. For DS determination, accurately pre-weighed (5 g) carboxymethylated starch was dispersed in 150 mL acetone, and then 15 mL (5 M HCl) were mixed with the suspension and agitated for 30 min. In this step, the Na-CMS was converted into H-CMS (protonated CMS). The product was rinsed with 80% methanol multiple times until the pH became neutral. The mixture was filtered, immersed in acetone, and stirred for a further 15 min. Finally, the dispersion was filtered and dried for 24 h at 50 °C. A total of 2 g of CMS were dissolved in 1% (*w*/*v*) sodium chloride solution and titrated with 1 M NaOH. The DS was evaluated using the following equations.

(1) $$DS=\frac{M_{o}\times n_{NaOH}}{m_{c}-n_{NaOH}\times M_{R}}$$

(2) $$m_{c}=m_{p}-\left[\frac{m_{p}\times F}{100}\right]$$

where *DS* is the degree of substitution; *M_o_* represents the molar mass of the AGU (g/mol); *M_R_* is the molar mass of carboxymethyl residue (g/mol); n_NaOH_ is the amount of NaOH used (mol); *m_p_* is the weight of the polymer taken (g); *m_c_* is the corrected weight of the polymer (g); and *F* is the percentage of moisture.

### 2.4. Characterization

To confirm the required modification, three different characterization techniques including Fourier-transform infrared spectroscopy (FTIR), X-ray diffraction, and thermogravimetric analysis (TGA) were used.

To analyze the chemical structure of native and carboxymethylated starches, the dried specimens with certain amounts were mixed with KBr and pressed into a transparent disk. The FTIR analysis was carried out using the FTIR spectroscopy instrument (Perkin Elmer, Waltham, MA, USA, Model: FTIR Frontier). Samples were analyzed by scanning the wavelength between 4000 and 400 cm^−1^ to obtain the percentage of absorbance. The infrared spectroscopy detected the vibration characteristics of chemical functional groups in the samples. Likewise, the X-ray diffraction pattern of native tapioca starch and its carboxymethylated form were recorded with the XRD instrument (Model PAnalytical Xpert Powder, PANalytical, Almelo, Netherlands). The XRD is a fast analytical method for identifying phases in crystalline materials. The operating conditions were maintained at 40 kV and 40 mA. Scanning was performed from 5 to 80° (2θ). The XRD analysis software (X’Pert HighScore Plus) was utilized to obtain the crystallinity of the NTS and CMTS. The thermal stability of the modified starch was quantified with a thermogravimetric analysis instrument (TGA; model: Perkin Elmer, Simultaneous Thermal analyzer, Model: STA 6000, Waltham, MA, USA) in the nitrogen atmosphere. It is a significant design parameter when selecting drilling mud additives. During testing, 5 mg of powdered starch sample was loaded in an alumina container followed by heating at a heating rate of 10 °C/min under N_2_ at 20 mL/min from 30 °C to 800 °C.

### 2.5. Experimental Design

Design Expert software v 13.0.1.0 (StatEase, Minneapolis, MN, USA) was used in this work to create the experimental design and analyze the results using the response surface methodology (RSM). The RSM is a user-friendly statistical tool for designing, analyzing, optimizing, and validating experimental findings [20]. An optimum (custom) design has been used to examine the influence of selected factors on fluid rheology, establish a mathematical correlation between the selected factors and responses, and explain the interactions between the factors. In this work, CMTS concentration and temperature were considered the factors, while PV and YP were selected as responses. The level of factors and the corresponding codes of different independent variables are given in Table 1.

The process performance was assessed by evaluating the responses based on the input factors *X*_1_, *X*_2_, …, *X_n_*. The general relationship between response parameters and input process parameters is described by:

(3) $$Y=f\left(X_{1},X_{2,},\;X_{3},\;\ldots X_{n}\right)+error$$

where *f* represents the real response function, and error describes the differentiation.

The quadratic equation model of the response Y derived as a function of the levels of independent variables expressed according to Equation (4) was used to predict optimal conditions. It is a second-order polynomial equation model for predicting the optimum point between factors and responses [21].

(4) $$Y=\beta_{o}+\sum_{i=1}^{k}\beta_{i}x_{i}+\sum_{i=1}^{k}\beta_{ii}x_{i}{}^{2}+\sum_{t_{i}=j}^{k}\sum_{j}^{k}\beta_{ij}x_{i}x_{j}+\ldots e$$

where *β* is the regression coefficient; *i* and *j* are the linear and quadratic coefficients, respectively; *k* represents the number of examined factors and optimized by the experiment, while *e* represents the random error.

Based on the selected design, thirty (30) experimental runs were performed for rheological analysis. Randomized experiments were carried out to eliminate errors caused by systematic patterns in the variables. Moreover, each experimental run was carried out three times, and an average of the results was reported.

### 2.6. Formulation of Mud Samples

The mud samples were formulated considering the API recommended practices. The muds were prepared according to the experimental design described in the previous section. The mud additives were mixed using a high-speed Fann Multimixer to prevent the degree of lumps formation within the mud system. A mixer cup was filled with 350 mL of deionized water followed by adding 1.5 ppb of xanthan gum (XG). The product was mixed at 11,500 rpm for 20 min to ensure a homogenous mixture. The pH was maintained at the API recommended range (9.5–10) by adding sodium hydroxide. The modified starch and polyanionic cellulose (PAC) as a rheology enhancer as well as a fluid loss control agent were added and mixed again for 20 min. Finally, 115 ppb of calcium carbonate was added to the mixture to achieve the required mud weight. The mixture was mixed for a further 20 min. The additives used in the mud preparation are summarized in Table 2.

### 2.7. Thermal Stability of Mud Samples

To examine the thermal stability of the formulated muds, the prepared samples were kept for 16 h in a hot roll oven (Fann roller oven) at selected temperature conditions (180 °F, 210 °F, and 240 °F). It provided an excellent approach of aging drilling mud samples at higher temperatures. The aging cells containing mud samples were pressurized to 1000 psi pressure and were subjected to the defined temperature while agitating on power driven rollers. After the hot rolling, the rheological properties were tested and recorded.

### 2.8. Rheological Measurements

The drilling fluid’s rheological properties influence a variety of drilling parameters including cuttings carrying capacity, hole cleaning, and pressure drop control. These are the most important aspects monitored throughout the drilling process. Thus, extra attention should be paid while determining these parameters. The rheological properties of all the fluid samples were evaluated in compliance with the American Petroleum Institute (API) standards using the Fann V-G meter 35SA [22]. Equations (5) and (6) were used to calculate the plastic viscosity and yield point of the drilling fluid.

(5) $$\begin{array}{c} PV=R_{600}-R_{300} \end{array}$$

(6) $$\begin{array}{c} YP=R_{300}-PV \end{array}$$

where *R*_600_ and *R*_300_ are the dial readings at 600 and 300 rpm, respectively. *PV* and *YP* are the plastic viscosity (cP) and yield point (lb/100 ft^2^), respectively.

Shear stresses and viscosities were computed at two rotational speeds of 600 and 300 rpm, which correspond to a shear rate of 1021.38 and 510.67 s^−1^, respectively. To obtain a homogeneous fluid, the samples were agitated for 5 min before performing each experimental run.

### 2.9. Rheological Modeling and ANOVA Analysis

The rheology of a drilling fluid is dictated by the fluid’s shear stress and shear rate relationship. To analyze which model is most suitable for the drilling muds, the shear stress over a range of shear rates was measured using the Discovery Hybrid Rheometer (Model: DHR-1). The rheological properties of the mud samples were evaluated, and the obtained results were used to evaluate their fitness with the conventional models. In this study, Bingham plastic, Power law, and Herschel–Bulkley models were employed to study the shear thinning tendency of the formulated muds. In addition, regression equations have been obtained by applying RSM for rheological properties, namely PV and YP. In order to explore the influence of selected variables and their interactions on responses, the obtained models were analyzed to test their adequacy by using ANOVA. Finally, the system was optimized and validated by further experiments to obtain the optimum rheological parameters values for a bentonite-free mud system. R^2^ values for all the developed models were used to analyze the quality of fit. The equations derived from a regression analysis of experimental results were applied to develop response surfaces and were reported. The model’s statistical summary is given in Table 3.

## 3. Results and Discussion

### 3.1. Degree of Substitution (DS) and Reaction Efficiency (RE)

**Figure 1:**
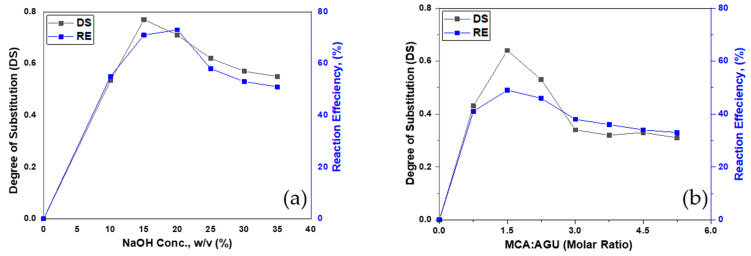
Effect of (**a**) NaOH concentration and (**b**) MCA molar ratio on DS and RE of CMTS.

In this study, two parameters were examined including the sodium hydroxide concentration and MCA molar ratio. Starch carboxymethylation was conducted at varying aqueous sodium hydroxide concentrations ranging from 10 to 35% (*w*/*v*). It was used in the process to initiate the reaction. Figure 1a demonstrates the influence of the NaOH concentration on DS and RE. The DS value was observed to improve dramatically as the NaOH concentration rose to 15% (*w*/*v*) with a maximum substitution degree of 0.77 and reaction efficiency of 71%. A higher concentration reaction with NaOH facilitates the swelling of starch granules and develops more starch-O-Na, culminating in a much greater risk of carboxymethylation in the starch molecules with the OH group. However, during carboxymethylation, it was observed that the reaction efficiency decreased above 15% of the NaOH concentration due to the dominant side reaction of NaOH with MCA. The degree of the substitution value was then lowered by raising the NaOH concentration above 15%. Similar findings were also reported for sago starch carboxymethylation by Yaacob, et al. [23]. Two main approaches occurred concurrently during the process including etherification and sodium glycolate formation. In the etherification, the starch reacted with MCA in the presence of NaOH, and the side reaction occurred between NaOH and MCA that produced sodium glycolate, which reduced both DS and RE values.

Figure 1b highlights the impact of the MCA ratio to AGU (0.75–5.25) on the values of DS and RE. In the current work, the highest value of DS was recorded as 0.64 with the MCA:AGU value of 1.5. The DS value decreased with the increase in the above ratio. The reason could be the side reaction of MCA and NaOH which resulted in the formation of sodium glycolate. The similar trend was observed for RE with about 49% at a molar ratio of 1.5. The RE decreased to 33% with the molar ratio of 5.25.

### 3.2. Characterization

#### 3.2.1. FTIR Analysis

**Figure 2:**
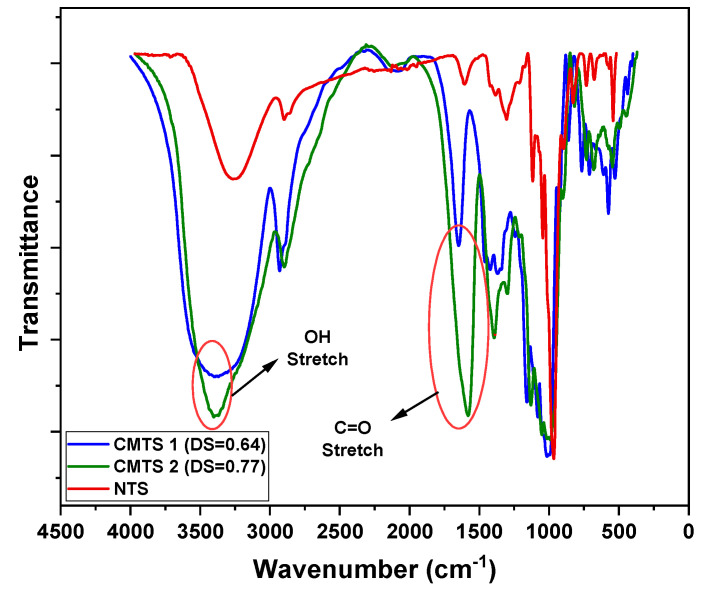
FTIR Spectra of NTS, CMTS 1, and CMTS 2.

Starch carboxymethylation has resulted in the emergence of new bands and has affected the intensity of native starch characteristics. The measurements confirmed the desired modification performed on the starch. The FTIR spectra of native and modified starch (DS 0.64 and 0.77) are shown in Figure 2. Since the number of peaks are less than 10, thus the material is not considered a complex material. A broad band at 3000–3600 cm^−1^ was assigned to O-H stretching, and it is due to hydrogen-bonded hydrogels of the polysaccharide chains. The intensity of this band also increased with the increase in the DS value. A band at 2933 cm^−1^ was attributed to CH_2_ symmetrical stretching vibrations. Similarly, the peaks at 2155 cm^−1^ were attributed to the absorption band of C≡C. In the double bond region (1500–2000 cm^−1^), the prominent absorption peaks for asymmetric and symmetric COO- vibrations were found in the carboxymethyl starch spectra at about 1648 and 1611 cm^−1^. This result is in agreement with the study of Liu, et al. [24]. This bond confirmed that the -OCH_2_COO- group was introduced into the starch molecule. This carbonyl double bond indicates the presence of a carboxyl group in the studied sample. These bands confirmed the carboxymethylation modification of the native tapioca starch.

#### 3.2.2. XRD Analysis

**Figure 3:**
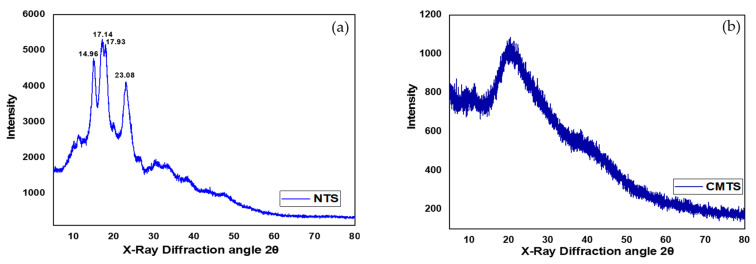
X-ray Diffractograms of (**a**) Native Tapioca Starch (NTS) and (**b**) Carboxymethylated Tapioca Starch (CMTS).

The crystalline structure of the carboxymethylated tapioca starch (CMTS) was investigated with XRD measurements. X-ray diffractograms of native and modified tapioca starch samples are presented in Figure 3a,b, respectively. A typical C-type crystallinity pattern was shown in the diffractogram of tapioca starch. This pattern is in agreement with the literature [25,26]. The crystallinity was significantly decreased after carboxymethylation. This reduction in crystallinity may be either related to the alkaline condition or due to the rupture of starch granules due to the presence of water. The effect of alkaline conditions during the modification opens up the potential use of carboxymethyl starches as being super absorbent because amorphous granules can increase the absorption of water. Due to the combined effect of water and heat treatment, the rupture of starch particles causes the breaking of chemical connections in starch molecules. The pattern has strong reflections at 14.96, 17.14, 17.93, and 23.08° of the diffraction angle (2θ) for the native starch. After chemical modification (Figure 3b), the visible reflections disappeared, revealing that the crystalline characteristics of the starch particles had been abolished.

#### 3.2.3. Thermogravimetric Analysis

**Figure 4:**
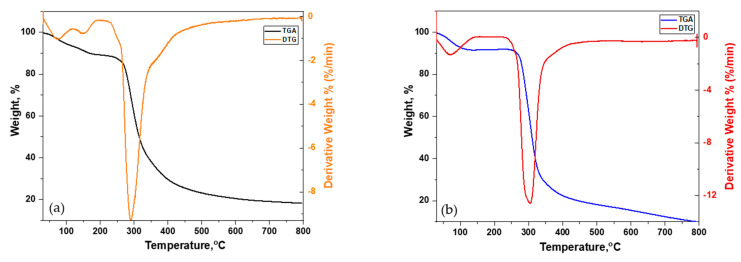
Thermograms of (**a**) Native Tapioca Starch (NTS) and (**b**) Carboxymethylated Tapioca Starch (CMTS).

The thermal stability of NTS and CMTS was determined by thermogravimetric analysis (TGA). TGA and DTG curves for NTS and CMTS are presented in Figure 4a,b. The thermal stability of polysaccharides such as starch and starch-based compounds is influenced by their composition, chemical structure, and storage environments. For both NTS and CMTS, different weight loss stages can be observed. The weight loss starting from 50 °C to 120 °C is different because of the different moisture contents. This stage is solely based on the quantity of water existing in the respective starch and has no influence on the starch’s overall thermal stability. The next degradation stage begins to decrease, which corresponds to the starch structural decomposition. In the majority of cases, the main component that is lost below 300 °C is moisture content; however, if not properly washed, the starch may have other impurities and may be lost before 300 °C. Heating to 500–600 °C yields carbonized material and ash.

In the current study, for NTS, the first weight loss (≈6%) commences around 30–70 °C and may be because of the residual solvents which are left after washing. The second weight loss starts at 70 °C and ends at 160 °C, corresponding to 4% which is because of the moisture content and the loss of water from starch. It is due to the dehydration reaction between the hydroxyl groups in NTS. The third and main degradation stage starts at a range of 250–320 °C. The DTG curves also showed the maximum weight loss at about 300 °C for native starch.

The TGA and DTG curves for CMTS show two degradation stages; the first weight loss at around 30–130 °C is because of water, and the second weight loss at 250–365 °C corresponds to the degradation and decomposition of the starch structure. The first weight loss corresponds to a total of 20%, and the second weight loss is 60%. It is worth mentioning that the maximum weight loss for CMTS starch has shifted to a higher temperature (above 300 °C), which shows that the thermal stability of starch has increased after carboxymethylation. Similar observations have been found by other authors, Ren, et al. [27]. In native starch, it can be believed that the primary decomposition mechanism is via the dehydration reaction between the hydroxyl groups.

### 3.3. Rheological Parameters and RSM Study

**Figure 5:**
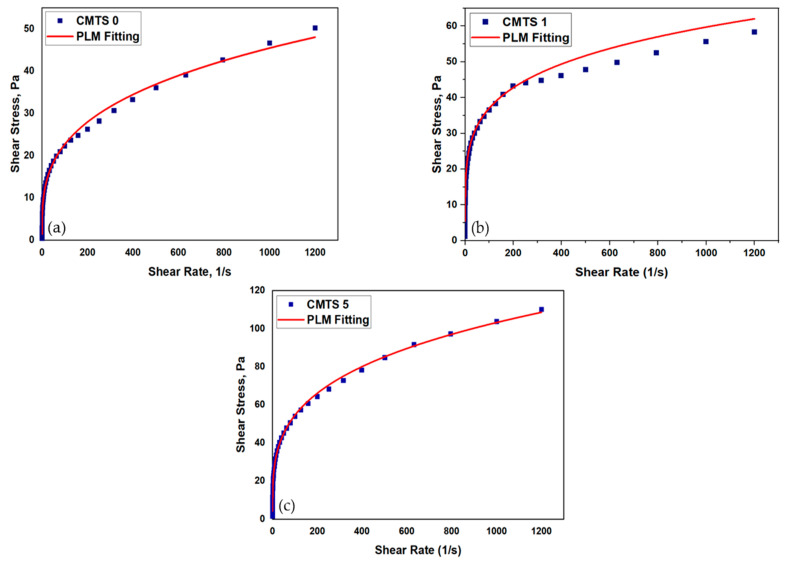
Rheograms of (**a**) CMTS 0, (**b**) CMTS 1, and (**c**) CMTS 5 mud sample.

It is vital to understand the correlation between shear stress and the shear rate in order to analyze the behavior of a drilling mud and its potential to suspend and carry drilled cuttings. The development of an appropriate rheological model is essential for a comprehensive description of the rheological properties of drilling muds. Drilling fluids are non-Newtonian in nature, showing a non-linear relationship between shear rate and shear stress. In this work, three mud blends including CMTS 0 (Control), CMTS 1, and CMTS 5 were selected for rheological modeling. In the first blend, no CMTS was added, while in CMTS 1 and CMTS 5, the concentrations of 1 ppb and 5 ppb were added. The rheometer readings in terms of shear stress and shear rate were plotted and are presented in Figure 5a–c. The figures show a nonlinear relationship between shear stress and the shear rate. After comparing the rheograms, it was found that the shear stress values of the CMTS 5 sample were higher than those of CMTS 0 and CMTS 1. This is due to the increased flow resistance caused by the higher starch content in the mud.

The obtained modeling parameters of each model are given in Table 4. From the obtained data, it was concluded that all studied samples’ data were best fitted with the Power law model (PLM) with R^2^ values of 0.99, 0.97, and 0.99 for CMTS 0, CMTS 1, and CMTS 5, respectively. The flow behavior indices for the samples were 0.30, 0.23, and 0.21, while the consistency indices were 5.6, 12.2, and 15.12 for CMTS 0, CMTS 1, and CMTS 5, respectively. From the obtained data, it was noticed that the value of the flow behavior index was reduced with an increase in the CMTS concentration. On the other hand, the introduction of starch enhanced the consistency index of the blends.

None of the studied mud samples showed an agreement with the Bingham plastic model because all the blends showed the R^2^ value to be less than 0.80 when fitted with the Bingham model. The *n* and k values for all the mud blends showed almost the same behavior of pseudoplasticity (Table 4).

### 3.4. Model Fitting and Statistical Analysis

Different models (linear, 2FI, quadratic, cubic, and quartic models) were fitted with the experimentally obtained data to opt for the most appropriate regression equations. In Table 5, the efficacy of each model is given in terms of various parameters. Both the R^2^ and *p*-value were taken into consideration for the selection of the most suitable model for the studied responses. According to this criterion, the model would not be acceptable if the R^2^ value is close to one, whereas the *p*-value is not significant and vice versa. Based on the above criterion, the best statistical models were quadratic and cubic for PV and YP, respectively. Similarly, the lack of fit was insignificant for both models.

The model selection has been made as suggested by the RSM tool. In terms of coded factors, the obtained final equations are given below:

(7) $$PV=16.2+5.96X_{1}-3.33X_{2}+0.291X_{1}X_{2}+0.558X_{1}{}^{2}-0.61BX_{2}{}^{2}$$

(8) $$\begin{array}{c} \begin{array}{c} YP=26.5+12.5X_{1}-18X_{2}-7.09X_{1}X_{2}+1.35X_{1}{}^{2}+3.96X_{2}{}^{2}-0.658X_{1}{}^{2}X_{2}-\\ 1.46X_{1}X_{2}{}^{2}+1.1X_{1}{}^{3}+1.45X_{2}{}^{3} \end{array} \end{array}$$

where *X*_1_ and *X*_2_ represent the CMTS concentration and temperature, respectively.

The model adequacy was further evaluated through an analysis of variance. Here, the quadratic and cubic regression coefficients, as well as the correlation between the variables in the model, were calculated. At a *p*-value (probability of error value) of <0.05, each independent factor’s effect was considered significant. The *p*-value represents each regression coefficient’s significance (i.e., the interaction effect of each cross product). The significance of the regression coefficient is inversely related to the *p*-value. Table 5 summarizes the ANOVA details for the model’s regression. For both models, the lower error probability value indicated that the generated models were statistically significant for describing the experimental results.

It is considered that the model with a higher coefficient of determination value, “R^2^”, can have high levels of multicollinearity which validates the regression models. In the current work, the standard deviation and coefficient of variation of the model were insignificant; thus, the models were considered adequate to predict the rheological properties of the formulated mud, with an acceptable adjusted R^2^ value. The R^2^ and adjusted R^2^ values are given in Table 6.

Here, for the plastic viscosity, the predicted R^2^ of 0.896 is in close agreement with the adjusted R^2^ of 0.922, suggesting that the difference is <0.2. Besides, the adequate precision which measures the signal to noise ratio for plastic viscosity was found to be 28.88, which is higher than 4, which is desirable. Hence the generated model can be applied to navigate the design space. Likewise, for the yield point, the predicted R^2^ of 0.947 is in reasonable agreement with the adjusted R^2^ of 0.983; i.e., the difference is <0.2. Moreover, the adequate precision for the yield point was reported to be 42.04. Thus, the generated model for YP can be considered appropriate for the design space. These findings were in close agreement with the results reported by Betiha, et al. [28].

**Figure 6:**
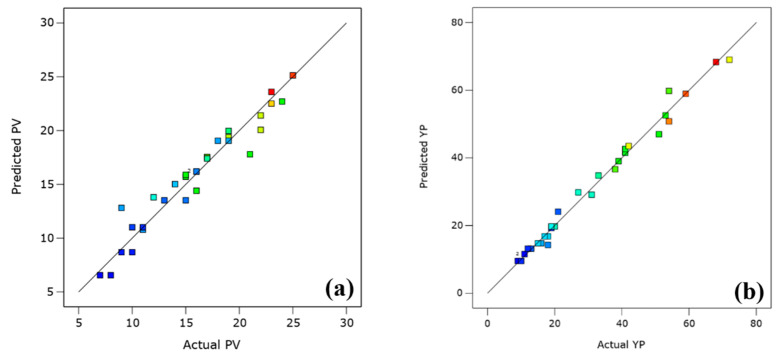
Predicted versus actual plots of (**a**) PV and (**b**) YP.

Figure 6a,b illustrates the plot of the predicted versus actual values of the responses. The predicted values were uniformly and closely distributed to the actual responses and showed a reasonable agreement (R^2^ = 0.93). This showed that the generated regression models can efficiently describe the relationship between the factors and the responses in the studied range. Since the data points are uniformly spread close to or on a straight line, the error is thus negligible within the operational parameter boundaries.

**Figure 7:**
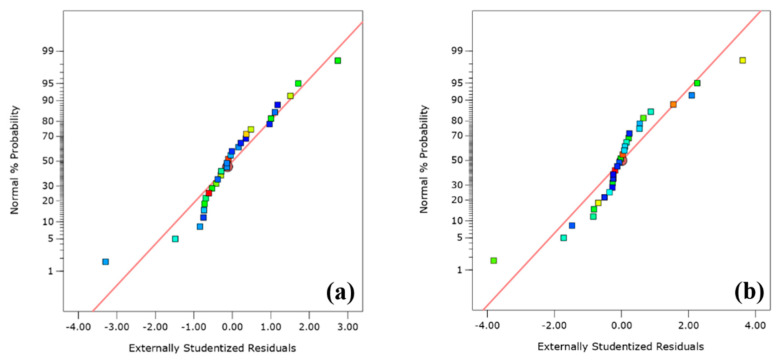
Normal probability plots of the residuals (**a**) PV and (**b**) YP.

**Figure 8:**
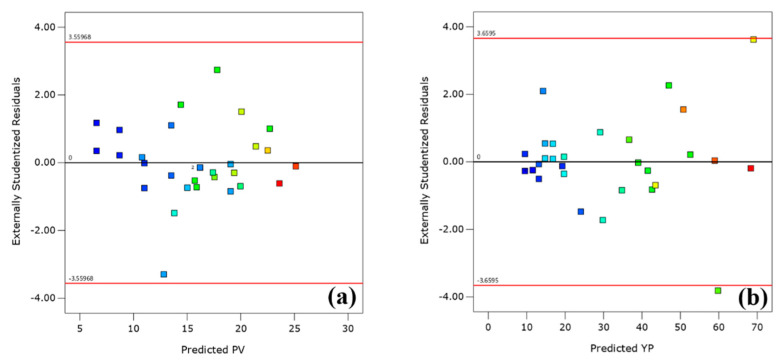
Plot of the residuals vs. predicted responses of (**a**) PV and (**b**) YP.

The adequacy of the generated models was also investigated using residuals. It is the difference between the observed and predicted responses. This analysis was accomplished using the normal probability plots as well as residual vs. predicted plots. Figure 7a,b demonstrates that the errors are normally distributed in a straight line in the normal probability plots of the residuals and are considered trivial. In addition, the plots of residuals vs. predicted responses (Figure 8a,b) are less organized; they depict that both models are appropriate, and there is no infringement of the concept of independence or continual variance in any model.

**Figure 9:**
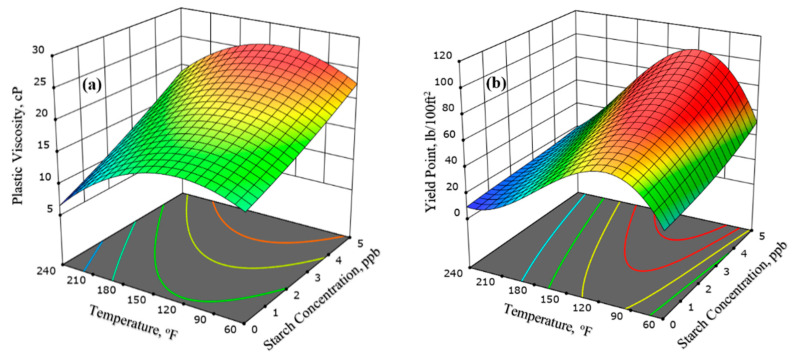
3D surface plots of (**a**) Plastic viscosity and (**b**) Yield point of mud.

Plastic viscosity and the yield point are believed to be the most essential rheological parameters of drilling muds. The CMTS concentration was observed to influence the performance of the drilling mud formulations significantly. Figure 9 presents the 3D response surface plots that show the interaction effect on the plastic viscosity in terms of the CMTS concentration and temperature. In Figure 9a, it was determined that the plastic viscosity was reduced with an increase in the hot roll temperature, demonstrating an inverse relationship. This decline is due to the partial thermal degradation of the xanthan gum and starch in the mud. However, the PV was found to be maximum with the highest CMTS concentration used in this study at a temperature of 180 °F. Below 180 °F, the increase in the CMTS concentration also increased the PV values, showing no adverse effect of temperature on the modified starch in this range. This increase in PV values is attributed to the fact that the starch granules become soluble in the mentioned range, which further aids in the increase in overall viscosity of the fluid by developing a gel-like structure with other additives. However, above 180 °F, the plastic viscosity showed an inverse relationship with the temperature. The combined effect of the CMTS concentration and temperature on PV is prominent in comparison with an individual effect. Furthermore, owing to the thermal degradation of the mud samples’ components, shear stress values reduced as the temperature rose for all the samples. All the obtained PV values were within the API’s suggested range when the modified starch was used as a potential additive. Since the minimum PV is often recommended in drilling operations in order to accelerate the rate of penetration (ROP), lower energy is required for mud circulation, and this provides efficient cooling and lubrication features for the downhole tools.

Similarly, the potential of the drilling mud to take the drilled cuttings from the annulus can be determined by the YP values, which should be fairly strong for the proper transport of the cuttings. It is an indicator of the drilling mud’s thixotropic characteristics at flow conditions, and its value can be regulated using different chemical additives. High YP values, however, produce additional pump pressure which should be prevented. Under flowing conditions, the yield strength is dependent on the electro-chemical charges in the drilling fluid. Figure 9b shows the combined effect of the CMTS concentration and temperature on the yield point of the mud blends. It was found that the yield point values are decreasing with an increase in temperature. An increased YP value was induced by the predominance of an attraction between the solid particles present in the mud. Conversely, a reduction in the yield point value was observed when repulsive forces prevailed. Since the yield point of WBM is proportional to its PV, thus the influence of temperature on the YP is identical to the influence of temperature on the PV. The maximum YP value in the current study was recorded at the highest CMTS concentration with a temperature of 180 °F. The YP value of the control mud at 240 °F was 7 lb/100 ft^2^, while at the same temperature, CMTS showed an improved YP of 19 lb/100 ft^2^. It is worth mentioning that the optimum YP values of the CMTS based mud blends remained in the range recommended by API standards.

### 3.5. Numerical Optimization and Confirmation

After evaluating the impact of each factor on the selected responses, numerical optimization was performed to generate a combination of factors using the software. The range of the factors (CMTS concentration and temperature) was set between lower and upper levels, which were coded to cover a broad range. All the factors were ranged, while a target for PV was set as 15 cP. Table 7 summarizes the input parameters.

The desirability function (D) was applied to define an appropriate solution to establish the optimal conditions. The overall desirability (D) is the geometric (multiplicative) mean of all individual desirabilities (*d_i_*) that range from 0 (least) to 1 (most). The following equation was used for the desirability function.

(9) $$\begin{array}{c} D={\left(\overset{n}{\underset{d=1}{\prod }}d_{i}\right)}^{\frac{1}{n}} \end{array}$$

where *n* is the number of responses.

**Figure 10:**
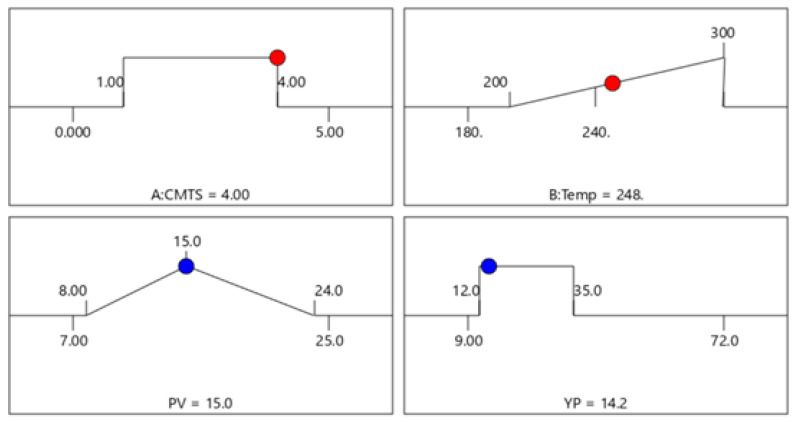
DOE Optimization solutions.

Using the DOE software, various sets of optimum operating variables and corresponding parameters were generated. To reflect the accuracy between the experimental findings and suggested solutions, the desirability of the optimal solutions was found to be 0.70. The parameters’ optimization solutions are given in Figure 10 and Table 8.

Confirmation of the models was performed by conducting extra experiments by selecting the new CMTS concentration and temperature. The CMTS concentration and temperature were set to 3.5 ppb and 250 °F, respectively. Three extra experimental runs were performed, and the obtained data are presented in Table 9.

All the obtained results showed a good agreement with the predicted mean which showed the accuracy of the generated models.

Overall, the rheological properties in terms of plastic viscosity and the yield point were improved by utilizing the modified starch. The improvement was due to the enhanced solubility and thermal stability at higher temperature conditions. The emergence of new groups improved the suspension capabilities of the bentonite free mud system. In addition, such muds could improve the rate of penetration as well as the reduced formation damage which is caused due to the filtration of solids into the exposed formation. Furthermore, the generated empirical models were found adequate based on the insignificant *p* values and highest R^2^ values.

## 4. Conclusions

The current research demonstrated the applications of carboxymethylated tapioca starch for the non-damaging formulations of drilling mud at various temperature conditions. As a result of experiments conducted on the mud sample containing modified starch, it was concluded that the thermal stability of the native tapioca starch was enhanced with carboxymethylation. Sodium hydroxide (NaOH) and monochloroacetic acid (MCA) concentrations have a significant effect on the modification of starch granules. FTIR analysis confirmed the modification by showing new bands of carboxymethyl group. The crystallinity of the samples was reduced after carboxymethylation when compared with the native starch. The thermal stability of starch was enhanced after modification, resulting in a better performance at higher temperatures with acceptable rheological properties. The mud formulated with modified starch showed shear thinning behavior, and shear stress was increased with the addition of the CMTS concentration into mud. The plastic viscosity and yield stress of mud blends showed a negative relationship with temperature which was due to the degradation of additives when exposed to high temperatures. The mud blends were tested for conventional rheological models which showed best fitting with the Power law model having the R^2^ value of >0.90. The generated mathematical models for plastic viscosity and yield point showed an adequate behavior with a high R^2^ value and insignificant *p*-value. The quadratic and cubic models were found the most suitable for the current ranges based on the lowest *p*-value and highest R^2^ values. It could be noted that the muds formulated with the CMTS additive performed better by replacing bentonite and other solid materials. Therefore, it is recommended that CMTS should be used to prepare drilling mud in terms of its effectiveness and green nature in higher temperature conditions.

## Figures and Tables

**Table 1:** Factor’s design and corresponding codes.

Factor	Unit	Codes	Levels	
			−α	+α
CMTS Conc.	ppb	X_1_	0	5
Temp	^o^F	X_2_	70	240

**Table 2:** Mud Composition.

Additive	Dosage/Conc.	Function
Deionized Water, mL	350	Continuous phase
Xanthan Gum, ppb	1.5	Primary viscosifier
Polyanionic Cellulose (PAC), ppb	2.0	Fluid loss control
Carboxymethylated Tapioca Starch (CMTS), ppb	0–5	Fluid loss control
Sodium Hydroxide (NaOH), ppb	0.5–0.75	pH control
Calcium Carbonate (CaCO_3_), ppb	115	Plugging material

**Table 3:** Summary of various models and suggested models for each response.

Source	Sum of Squares	df	Mean Square	F-Value	*p*-Value	Std. Dev.	R^2^	Adjusted R^2^	Predicted R^2^
**Plastic Viscosity**									
Mean	7552	1	7552						
Linear	609	2	304	51.1	<0.0001	2.44	0.791	0.776	0.750
2FI	5.25	1	5.25	0.879	0.357	2.45	0.798	0.775	0.749
**Quadratic**	**106**	**2**	**52.9**	**25.6**	**<0.0001**	**1.44**	**0.935**	**0.922**	**0.896**
Cubic	14.1	4	3.53	1.99	0.135	1.33	0.954	0.933	0.873
Quartic	9.32	4	2.33	1.42	0.272	1.28	0.966	0.938	0.806
**Yield Point**									
Mean	28954	1	28954						
Linear	7665	2	3832	41.4	<0.0001	9.62	0.754	0.736	0.701
2FI	169	1	169	1.89	0.181	9.47	0.771	0.744	0.709
**Quadratic**	**1738**	**2**	**869**	**35.2**	**<0.0001**	**4.97**	**0.942**	**0.930**	**0.902**
Cubic	472	4	118	19.6	<0.0001	2.45	0.988	0.983	0.947
Quartic	43.2	4	10.8	2.24	0.110	2.19	0.992	0.986	0.868

**Table 4:** Rheological Modeling Parameters.

Model	Power Law Model (PLM)		
Parameters	*τ = Kγ^n^*		
Plot	CMTS 0	CMTS 1	CMTS 5
K	5.63938 ± 0.25857	12.24214 ± 2.05839	15.121 ± 0.399
*n*	0.30221 ± 0.00641	0.23036 ± 0.01797	0.2173 ± 0.003
R-Square	0.99718	0.97257	0.99904
Adj. R-Square	0.99706	0.97145	0.99901

**Table 5:** Summary of ANOVA for model’s regression.

Source	Sum of Squares	df	Mean Square	F-Value	*p*-Value	
**Plastic Viscosity (Quadratic Model)**						
Model	720	5	144	69.6	<0.0001	Significant
X_1_-CMTS	443	1	443	214	<0.0001	
X_2_-Temp	194	1	194	93.6	<0.0001	
X_1_ X_2_	5.25	1	5.25	2.54	0.124	
X_1_^2^	1.49	1	1.49	0.719	0.405	
X_2_^2^	104	1	104	50.4	<0.0001	
**Yield Point (Cubic Model)**						
Model	10043	9	1115	186	<0.0001	Significant
X_1_-CMTS	257	1	257	42.9	<0.0001	
X_2_-Temp	3258	1	3259	543	<0.0001	
X_1_ X_2_	409	1	409	68.1	<0.0001	
X_1_^2^	7.74	1	7.74	1.29	0.270	
X_2_^2^	68.4	1	68.4	11.4	0.00302	
X_1_^2^ X_2_	9.14	1	9.14	1.52	0.232	
X_1_ X_2_^2^	277	1	277	46.1	<0.0001	
X_1_^3^	1.60	1	1.60	0.267	0.611	
X_2_^3^	184	1	184	30.6	<0.0001	

**Table 6:** R^2^ and adjusted R^2^ values of the models.

Parameters	PV	YP
R^2^	0.935	0.988
Adjusted R^2^	0.922	0.983
Predicted R^2^	0.896	0.947
Adeq. Precision	28.88	42.04

**Table 7:** Rheology optimization input parameters.

Parameters		Goal	Lower Limit	Upper Limit
Factors	CMTS	In range	1	4
	Temp	In range	200	300
Responses	PV	Target = 15	8	24
	YP	In range	12	35

**Table 8:** Rheological parameters in optimal conditions.

CMTS Conc.	Temp	PV	YP
4.00	248	15.0	14.2

**Table 9:** Confirmation of Model.

Response	Std Dev	Obtained Data Mean
PV	1.44	15.3
YP	2.45	17.3

## Data Availability

The data presented in this study are available on request from the corresponding authors.

## Nomenclature

WBM	Water based mud
CMS	Carboxymethyl starch
CMC	Carboxymethyl cellulose
NTS	Native tapioca starch
CMTS	Carboxymethyl tapioca starch
DS	Degree of substitution
API	American petroleum institute
HPHT	High pressure high temperature
TGA	Thermogravimetric analysis
XG	Xanthan gum
PAC	Polyanionic cellulose
TS	Tapioca starch
AV	Apparent viscosity
PV	Plastic viscosity
YP	Yield point
GS	Gel strength
HBM	Herschel Bulkley model
PLM	Power law model

## References

[1] Alcheikh I., Ghosh B. (2017). A Comprehensive Review on the Advancement of Non-damaging Drilling Fluids. Int J. Petrochem. Res..

[2] Ahmad H.M., Kamal M.S., Al-Harthi M.A. (2018). Rheological and filtration properties of clay-polymer systems: Impact of polymer structure. Appl. Clay Sci..

[3] Hamza A., Shamlooh M., Hussein I.A., Nasser M., Salehi S. (2019). Polymeric formulations used for loss circulation materials and wellbore strengthening applications in oil and gas wells: A review. J. Pet. Sci. Eng..

[4] Hamad B.A., He M., Xu M., Liu W., Mpelwa M., Tang S., Jin L., Song J. (2020). A Novel Amphoteric Polymer as a Rheology Enhancer and Fluid-Loss Control Agent for Water-Based Drilling Muds at Elevated Temperatures. ACS Omega.

[5] Zhang X., Jiang G., Xuan Y., Wang L., Huang X. (2017). Associating copolymer acrylamide/diallyldimethylammonium chloride/butyl acrylate/2-acrylamido-2-methylpropanesulfonic acid as a tackifier in clay-free and water-based drilling fluids. Energy Fuels.

[6] Cobianco S., Bartosek M., Lezzi A., Guarneri A. (2001). How to manage drill-in fluid composition to minimize fluid losses during drilling operations. SPE Drill. Completion.

[7] López O.V., Castillo L.A., Ninago M.D., Ciolino A.E., Villar M.A. (2017). Modified starches used as additives in enhanced oil recovery (EOR). Industrial Applications of Renewable Biomass Products.

[8] Harry T., Joel O., Ademiluyi F., Oduola K. (2016). Performance Evaluation of Local Cassava Starches With Imported Starch for Drilling Fluid. Am. J. Eng. Res..

[9] Harry T.F., Oduola K., Ademiluyi F.T., Joel O.F. (2017). Application of Starches from Selected Local Cassava (Manihot Exculenta Crantz) as Drilling Mud Additives. Am. J. Chem. Eng..

[10] Dankwa O., Appau P.O., Tampuri M. (2018). Performance evaluation of local cassava starch flour as a secondary viscosifier and fluid loss agent in water based drilling mud. Ghana Min. J..

[11] Su Z., Wang R., Liu J., Chen E. (2019). Filtration Reduction Mechanism of Environmental-Friendly Compound Modified Starch in Water-Based Drilling Fluids. Fresenius Environ. Bull..

[12] Bai X., Zhang X., Xu Y., Yong X. (2021). Synthesis and Characterization of Sodium Carboxymethyl Starch-Graft Acrylamide/1-Vinyl-2-Pyrrolidone Copolymers Via Central Composite Design and Using as Filtration loss Agent in Drilling Muds. Starch Stärke.

[13] Samavati R., Abdullah N., Hussain S. (2014). Rheological and fluid loss properties of water based drilling mud containing HCl-modified fufu as a fluid loss control agent. Int. J. Chem. Eng. Appl..

[14] Zoveidavianpoor M., Samsuri A. (2016). The use of nano-sized Tapioca starch as a natural water-soluble polymer for filtration control in water-based drilling muds. J. Nat. Gas. Sci. Eng..

[15] Elkatatny S. Assessing the Effect of Micronized Starch on Rheological and Filtration Properties of Water-Based Drilling Fluid. Proceedings of the SPE Middle East Oil and Gas Show and Conference.

[16] Sulaimon A.A., Akintola S.A., Johari M.A.B.M., Isehunwa S.O. (2020). Evaluation of drilling muds enhanced with modified starch for HPHT well applications. J. Pet. Explor. Prod. Technol..

[17] Minaev K.M., Martynova D.O., Zakharov A.S., Sagitov R.R., Ber A.A., Ulyanova O.S. (2016). Synthesis of Carboxymethyl Starch for increasing drilling mud quality in drilling oil and gas wells. Xx International Scientific Symposium of Students, Postgraduates and Young Scientists on Problems of Geology and Subsurface Development.

[18] Soto D., Leon O., Urdaneta J., Munoz-Bonilla A., Fernandez-Garcia M. (2020). Modified Starch as a Filter Controller in Water-Based Drilling Fluids. Materials.

[19] Rachtanapun P., Simasatitkul P., Chaiwan W., Watthanaworasakun Y. (2012). Effect of sodium hydroxide concentration on properties of carboxymethyl rice starch. Int. Food Res. J..

[20] Salehnezhad L., Heydari A., Fattahi M. (2019). Experimental investigation and rheological behaviors of water-based drilling mud contained starch-ZnO nanofluids through response surface methodology. J. Mol. Liq..

[21] Bandara P.C., Nadres E.T., Rodrigues D.F. (2019). Use of response surface methodology to develop and optimize the composition of a chitosan–polyethyleneimine–graphene oxide nanocomposite membrane coating to more effectively remove Cr (VI) and Cu (II) from water. ACS Appl. Mater. Interfaces.

[22] Api R. (2009). 13I–Recommended Practice for Laboratory Testing of Drilling Fluids.

[23] Yaacob B., Mohd A.M.C.I., Hashim K., Abu B.B. (2011). Optimization of Reaction Conditions for Carboxymethylated Sago Starch. Iran. Polym. J..

[24] Liu J., Chen J., Dong N., Ming J., Zhao G. (2012). Determination of degree of substitution of carboxymethyl starch by Fourier transform mid-infrared spectroscopy coupled with partial least squares. Food Chem..

[25] Katsumi N., Okazaki M., Yonebayashi K., Kawashima F., Nishiyama S., Nishi T. (2015). New proposal for “crystalline index” of starch. Sago Palm.

[26] Dome K., Podgorbunskikh E., Bychkov A., Lomovsky O. (2020). Changes in the Crystallinity Degree of Starch Having Different Types of Crystal Structure after Mechanical Pretreatment. Polymers.

[27] Ren J.-L., Sun R.-C., Peng F. (2008). Carboxymethylation of hemicelluloses isolated from sugarcane bagasse. Polym. Degrad. Stab..

[28] Betiha M.A., Mohamed G.G., Negm N.A., Hussein M.F., Ahmed H.E. (2020). Fabrication of ionic liquid-cellulose-silica hydrogels with appropriate thermal stability and good salt tolerance as potential drilling fluid. Arab. J. Chem..

